# A Fast Self-Calibration Method for Dual-Axis Rotational Inertial Navigation Systems Based on Invariant Errors

**DOI:** 10.3390/s24020597

**Published:** 2024-01-17

**Authors:** Xin Sun, Jizhou Lai, Pin Lyu, Rui Liu, Wentao Gao

**Affiliations:** Navigation Research Center, Nanjing University of Aeronautics and Astronautics, Nanjing 210016, China; sunxin27@nuaa.edu.cn (X.S.); lvpin@nuaa.edu.cn (P.L.); liuruizhx@nuaa.edu.cn (R.L.); gaowentao@nuaa.edu.cn (W.G.)

**Keywords:** dual-axis rotational inertial navigation system, system-level self-calibration, invariant errors, backtracking navigation, observability analysis

## Abstract

In order to ensure that dual-axis rotational inertial navigation systems (RINSs) maintain a high level of accuracy over the long term, there is a demand for periodic calibration during their service life. Traditional calibration methods for inertial measurement units (IMUs) involve removing the IMU from the equipment, which is a laborious and time-consuming process. Reinstalling the IMU after calibration may introduce new installation errors. This paper focuses on dual-axis rotational inertial navigation systems and presents a system-level self-calibration method based on invariant errors, enabling high-precision automated calibration without the need for equipment disassembly. First, navigation parameter errors in the inertial frame are expressed as invariant errors. This allows the corresponding error models to estimate initial attitude even more rapidly and accurately in cases of extreme misalignment, eliminating the need for coarse alignment. Next, by utilizing the output of a gimbal mechanism, angular velocity constraint equations are established, and the backtracking navigation is introduced to reuse sensor data, thereby reducing the calibration time. Finally, a rotation scheme for the IMU is designed to ensure that all errors are observable. The observability of the system is analyzed based on a piecewise constant system method and singular value decomposition (SVD) observability analysis. The simulation and experimental results demonstrate that this method can effectively estimate IMU errors and installation errors related to the rotation axis within 12 min, and the estimated error is less than 4%. After using this method to compensate for the calibration error, the velocity and position accuracies of a RINS are significantly improved.

## 1. Introduction

Inertial navigation systems (INSs) are widely used in various automated unmanned vehicles for their advantages, such as good autonomy and strong concealment [1]. The precision of inertial navigation parameters directly affects the performance of an INS. In addition to using high-precision inertial devices, rotation modulation techniques can be introduced to improve the accuracy of an INS. The accuracy of rotational inertial navigation systems (RINSs) can be improved several times when using sensors of the same level. RINSs can self-calibrate by rotating their own gimbal mechanism to excite error parameters, which is more convenient and effective for error calibration [2,3]. Moreover, as the manufacturing cost and volume of inertial devices decrease, RINSs are gradually being applied to various systems and equipment [4,5,6,7,8].

Inertial measurement units (IMUs) typically require high-precision turntable calibration before leaving the factory. However, the service life of an RINS is generally more than ten years. During this period, IMUs can experience changes in the nominal error parameters due to component aging, the deformation of structural components that support the inertial devices, and other factors [9,10]. If the original calibration parameters are used over time, it will lead to a decrease in the accuracy of the inertial navigation system, which can have a negative impact on operational efficiency. Therefore, it is necessary to periodically conduct the high-precision autonomous calibration of RINSs to ensure the system’s performance when in use. However, the existing calibration methods take too long to calibrate and cannot quickly self-calibrate.

Currently, calibration methods are divided into discrete calibration and system-level calibration [11]. The former method involves removing the inertial navigation system, performing calibration using specialized high-precision turntables, and then reinstalling the inertial navigation system. The process is intricate and may introduce new installation errors [12,13]. The latter method uses navigation errors as observation variables to achieve the optimal estimation of inertial navigation system calibration parameters. This method eliminates the need for specific calibration sites and high-cost equipment. RINSs can be conveniently self-calibrated without an external turntable by exciting error parameters through the rotation of their own gimbal mechanisms. A 30-position calibration scheme was proposed for a dual-axis RINS in reference [14], which could calibrate 21 IMU errors by periodically rotating the axis.

Traditional system-level calibration methods for RINSs typically focus on calibrating IMU errors, including zero bias, scale factor errors, and installation errors. Due to the introduction of the gimbal mechanism, the installation errors between the IMU and the rotation axis also need to be accurately calibrated. Reference [15] utilized the difference in attitude between the two RINSs to estimate installation errors, but it could not achieve the self-calibration of the RINS. Reference [16] proposed a system-level self-calibration method for a dual-axis RINS to address two types of installation errors. However, this method requires the knowledge of an accurate initial attitude.

Currently, the primary method for system-level calibration uses the extended Kalman filter (EKF) to estimate errors [17,18]. In the traditional EKF framework, state error definitions, such as position and velocity, only consider magnitude differences and ignore directional differences. When the initial misalignment error is significant, it can lead to inconsistencies in the defined error coordinates [19]. Furthermore, due to the influence of specific force terms in the system matrix, the EKF defined with a linear error state encounters the problem of inconsistent variance estimation. This leads to the filtering estimate of errors that were initially unobservable, causing significant deviations and overly optimistic covariance estimates. To address these issues, the error equation can be projected and transformed to adapt to different application environments. Reference [20] eliminated the need for the high-speed integration of specific forces in the calculation of the Kalman filter transformation matrix by transforming the velocity error, which improved the accuracy and stability of the system. Reference [21] proposed the state transformation extended Kalman filter (ST-EKF), whereby the specific force vector in the system error model is replaced by the nearly constant gravity vector in a geographical coordinate system, ensuring consistent error state definitions and resolving the variance estimation inconsistency problem. In view of the strong non-linearity and high-dimensional problems of the RINS calibration error equation, the filtering method of sampling the probability space is also widely used in system-level calibration [22,23].

In recent years, the Lie group and manifold theories have been applied to some inertial-based applications [24,25]. By representing the states and their errors in double-direct space-isometric groups $SE_{2}\left(3\right)$ , new group-based system equations and error equations have been designed [26]. If the system is a group affine, even when the attitude deviation is significant, the attitude can be obtained through a linear error model [27]. Based on the existing EKF framework, Barrau used the Lie group theory to construct navigation parameters, establishing a navigation parameter error equation based on invariant errors, and used an invariant extended Kalman filter (IEKF) to unify the initial alignment process, eliminating the need for coarse alignment [28]. Reference [29] studied the characteristics of $SE_{k}\left(3\right)$ and the specific mathematical expressions of left-invariant and right-invariant errors under various coordinate frames. The right-invariant EKF projects the gravity vector into the inertial frame and has similar properties to the ST-EKF. The left-invariant EKF eliminates the influence of navigation parameters, and the filter performance does not depend on the selected coordinate system. Reference [30] investigated an attitude estimation method using the $SE_{2}\left(3\right)-\mathrm{EKF}$ with consideration of constant drift of the gyro. Therefore, this paper represents navigation parameter errors as invariant errors, transforming a non-linear system into a linear system, thereby improving the accuracy of calibration in cases of large misalignment angles.

To reduce the calibration time and improve calibration accuracy, this paper designs a rotation scheme and utilizes the output of a gimbal mechanism to establish extended measurement equations, rendering all errors observable. Subsequently, the observability of the system is analyzed based on the piecewise constant system (PWCS) observability method and singular value decomposition (SVD) observability analysis. Finally, the paper introduces backtracking navigation [31,32], enabling the sensor data to be reused to enhance the estimation accuracy within a short period.

The rest of this paper is organized as follows. Section 2 introduces the mathematical foundations for expressing navigation states using $SE_{k}\left(3\right)$ . Section 3 presents the IMU error model, gimbal mechanism errors, and the navigation error state model based on $SE_{2}\left(3\right)$ , demonstrating that this system is a group affine. Section 4 describes the designed rotation scheme, backtracking scheme, angular velocity constraints (AVCs) during rotation, the establishment of the measurement equations, and the observability analysis of the system. Section 5 validates the proposed method through simulations and experiments. Section 6 provides a summary and outlines future prospects.

## 2. Mathematical Preliminaries and the Framework of the Proposed Method

In order to apply the Lie group theory [33] in RINS system-level self-calibration, this section introduces the mathematical preliminaries of matrix Lie group and Lie algebra to facilitate subsequent error modeling and formula derivation. The matrix Lie group $SE_{2}\left(3\right)$ is used to represent the extended pose as [34].

(1) $$SE_{2}\left(3\right)=\left\{T=\left[\frac{C}{0_{2\times 3}}|\frac{v\;p}{I_{2\times 2}}\right]\in R^{5\times 5}|\begin{array}{c} C\in SO(3)\\ v,p\in R^{3\times 1} \end{array}\right\}$$

where SO(3) is the special orthogonal group and C,v,p are the attitude rotation matrix, speed, and position, respectively. The inverse of T is provided by

(2) $$T^{-1}=\left[\frac{C^{T}}{0_{2\times 3}}|\frac{-C^{T}v-C^{T}p}{I_{2\times 2}}\right]\in SE_{2}(3)$$

The Lie algebra associated with $SE_{2}\left(3\right)$ is provided by

(3) $$se_{2}\left(3\right)=\left\{\Xi =\zeta^{\wedge }\in R^{5\times 5}|\zeta \in R^{9\times 1}\right\}$$

where

(4) $$\zeta^{\wedge }={\left[\begin{array}{c} \rho \\ \nu \\ \varphi \end{array}\right]}^{\wedge }=\left[\frac{\varphi \times }{0_{2\times 3}}|\frac{\nu \;\rho }{0_{2\times 2}}\right]\in R^{5\times 5},\varphi ,\nu ,\rho \in R^{3\times 1}$$

The Lie algebra and Lie group can be transformed into each other via exponential mapping Exp(•) and logarithmic mapping Log(•) as follows:

(5) $$T=\mathrm{Exp}\left(\zeta \right)=\left[\frac{\exp\left(\varphi \right)}{0_{2\times 3}}|\frac{J_{l}\left(\varphi \right)\nu \;J_{l}\left(\varphi \right)\rho }{I_{2\times 2}}\right]$$

(6) $$\zeta =\mathrm{Log}\left(T\right)=\left[\begin{array}{c} \varphi a\\ J_{l}^{-1}\left(\varphi \right)v\\ J_{l}^{-1}\left(\varphi \right)p \end{array}\right]$$

where

(7) $$\varphi =\cos^{-1}\left(\left(\mathrm{tr}\left(C\right)-1\right)/2\right)$$

(8) $$\exp\left(\varphi \right)=\cos\varphi I_{3\times 3}+\left(1-\cos\varphi \right)aa^{T}+\sin\varphi \left(a\times \right)$$

(9) $$J_{l}\left(\varphi \right)=\left(\sin\varphi /\varphi \right)I_{3\times 3}+\left(1-\left(\sin\varphi /\varphi \right)\right)aa^{T}+\left(\left(1-\cos\varphi \right)/\varphi \right)\left(a\times \right)$$

(10) $$J_{l}^{-1}\left(\varphi \right)=\left(\varphi /2\right)\left(\cot\varphi /2\right)I_{3\times 3}+\left(1-\left(\varphi /2\right)\left(\cot\varphi /2\right)\right)aa^{T}-\left(\varphi /2\right)\left(a\times \right)$$

φ=φa is the rotation vector, φ denotes the rotation angle, and a is the unit-length axis of rotation. (•×) denotes the skew-symmetric matrix operation. When φ is assumed to be small, then $\exp\left(\varphi \right)\approx I_{3\times 3}+\left(\varphi \times \right)$ , $J_{l}\left(\varphi \right)\approx I_{3\times 3}+\left(\varphi \times \right)/2$ , and $J_{l}^{-1}\left(\varphi \right)\approx I_{3\times 3}-\left(\varphi \times \right)/2$ .

**Figure 1:**
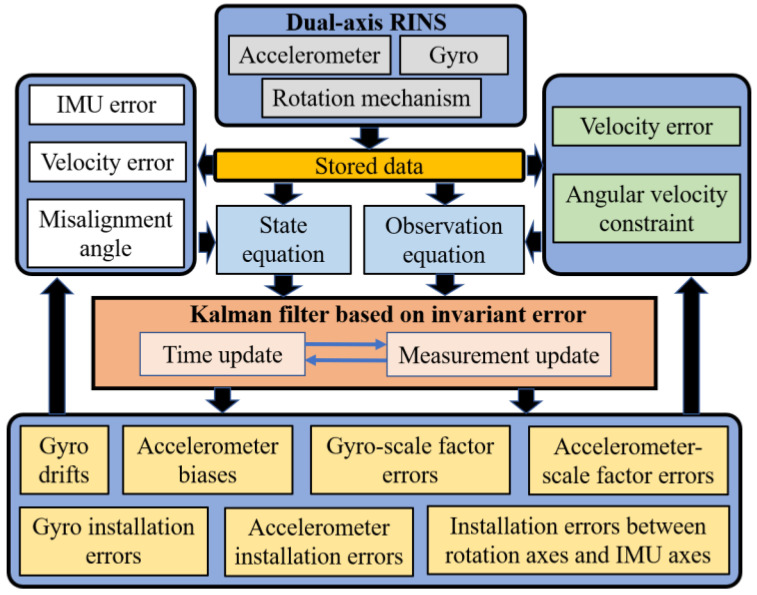
Schematic diagram of the proposed method in this paper.

In addition to the traditional IMU error, this article considers the installation error of the gimbal mechanism and stores the gyro, accelerometer, and gimbal mechanism signals outputs in real time. In order to quickly and accurately estimate the error parameters, the navigation parameters are established in the form of invariant errors, the linear Kalman filter is used to accurately estimate the calibration errors, and the stored data is reused through the backtracking algorithm to shorten the filter convergence time. Finally, a fast, autonomous, and accurate dual-axis rotation inertial navigation system-level self-calibration algorithm is achieved. The structure of the Kalman filter based on the invariant error during self-calibration is shown in Figure 1.

## 3. Gimbal Mechanism Installation Error Model, IMU Error Model, and Navigation Error Model

To better analyze the mechanism of the calibration process, several coordinate systems are defined in Table 1.

### 3.1. Gimbal Mechanism Installation Error Model

**Figure 2:**
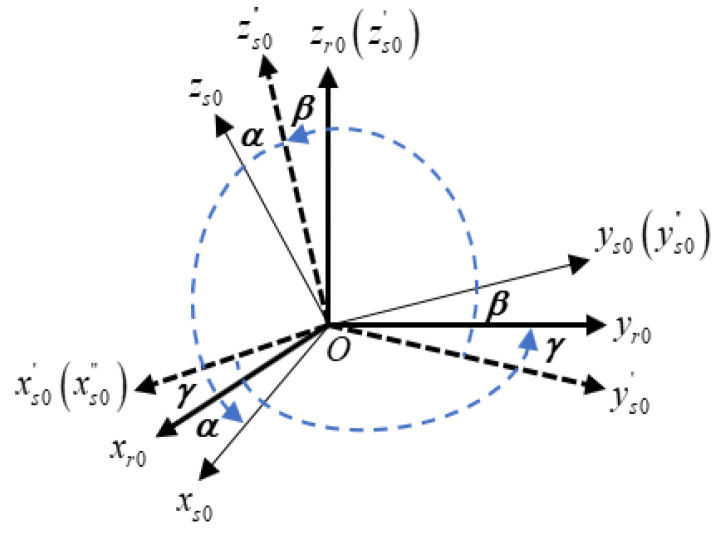
Definition of the s-frame and r-frame in the initial state.

For a dual-axis RINS, the IMU is installed on the dual-axis gimbal mechanism. Due to gimbal mechanism installation errors, the r-frame needs to be established to describe the spatial relationship between the r-frame and the s-frame. There is a transformation between the r-frame and the s-frame in the initial state, which is shown in Figure 2.

Generally, the gimbal mechanism installation error is small, and the transformation matrix between the r-frame and the s-frame is represented as:

(11) $$C_{r}^{s}=\left[\begin{array}{ccc} 1 & -\gamma & \beta \\ \gamma & 1 & -\alpha \\ -\beta & \alpha & 1 \end{array}\right]=I_{3\times 3}+\left(\mu \times \right)$$

where $\mu ={\left[\alpha ,\beta ,\gamma \right]}^{T}$ is the installation angle error between the s-frame and the r-frame.

### 3.2. IMU Error Model

**Figure 3:**
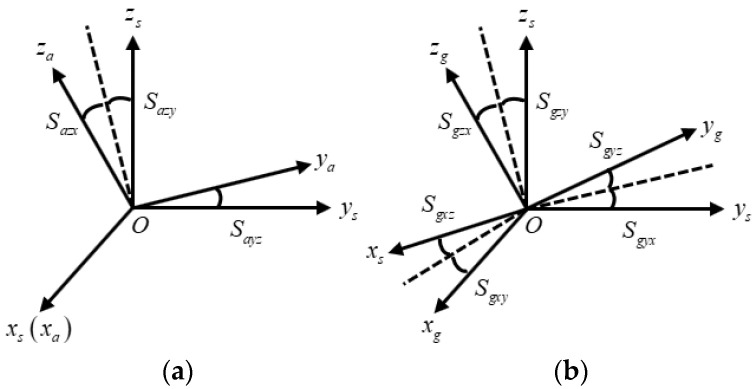
Definition of the sensor frame, accelerometer frame, and gyro frame. (**a**) Sensor frame and accelerometer frame; (**b**) sensor frame and gyro frame.

The coordinate system formed with the output of the three-axis accelerometer is the a-frame. The coordinate system formed with the output of the three-axis gyroscope is the g-frame. Due to the installation errors, the a-frame and g-frame are both non-orthogonal coordinate systems. Under normal circumstances, the a-frame and the g-frame are not coincident. Therefore, it is necessary to convert the three-axis acceleration output and the three-axis gyroscope output into the same sensor coordinate system, which is the s-frame. The definition of the s-frame is as follows [16]: $y_{s}$ is in the $x_{a}$ , $y_{a}$ plane, $x_{s}$ coincides with $x_{a}$ , while $z_{s}$ forms a right-handed orthogonal frame with $x_{s}$ and $y_{s}$ . The spatial relationships among the g-frame, a-frame, and s-frame are shown in Figure 3.

The IMU error model is defined in the s-frame. The gyros and accelerometers with errors are described as follows:

(12) $$\begin{array}{l} \omega_{is}^{s}=\left(I_{3\times 3}-C_{g}^{s}-K_{g}\right)\omega_{ig}^{g}-\varepsilon \\ f_{is}^{s}=\left(I_{3\times 3}-C_{a}^{s}-K_{a}\right)f_{ia}^{a}-\nabla \end{array}$$

where $\varepsilon ={\left[\varepsilon_{x},\varepsilon_{y},\varepsilon_{z}\right]}^{T}$ and $\nabla ={\left[\nabla_{x},\nabla_{y},\nabla_{z}\right]}^{T}$ are the constant gyro drifts and accelerometer biases. $\omega_{ig}^{g}={\left[\omega_{igx}^{g},\omega_{igy}^{g},\omega_{igz}^{g}\right]}^{T}$ and $f_{ia}^{a}={\left[f_{iax}^{a},f_{iay}^{a},f_{iaz}^{a}\right]}^{T}$ are the input of the gyro and accelerometer. $K_{g}$ and $K_{a}$ are the scale factor errors of the gyros and accelerometers. $k_{gi}\left(i=x,y,z\right)$ is the gyro-scale factor errors corresponding to the *X*g-axis, *Y*g-axis, and *Z*g-axis. $k_{ai}\left(i=x,y,z\right)$ is the accelerometer-scale factor errors corresponding to the *X*g-axis, *Y*g-axis, and *Z*g-axis. They are expressed as follows:

(13) $$K_{g}=\left[\begin{array}{ccc} k_{gx} & 0 & 0\\ 0 & k_{gy} & 0\\ 0 & 0 & k_{gz} \end{array}\right],K_{a}=\left[\begin{array}{ccc} k_{ax} & 0 & 0\\ 0 & k_{ay} & 0\\ 0 & 0 & k_{az} \end{array}\right]$$

$C_{g}^{s}$ , $C_{a}^{s}$ are the installation errors of the gyros and accelerometers. Through Figure 3, $C_{g}^{s}$ and $C_{a}^{s}$ can be expressed as follows:

(14) $$C_{g}^{s}=\left[\begin{array}{ccc} 0 & S_{gxz} & S_{gxy}\\ S_{gyz} & 0 & S_{gyx}\\ S_{gzy} & S_{gzx} & 0 \end{array}\right]=(S_{g}⊕)\;\;\;\;\;\;\;\;\;C_{a}^{s}=\left[\begin{array}{ccc} 0 & 0 & 0\\ S_{ayz} & 0 & 0\\ S_{azy} & S_{azx} & 0 \end{array}\right]=\left(S_{a}⊙\right)$$

where $S_{g}={\left[S_{gxz},S_{gxy},S_{gyz},S_{gyx},S_{gzy},S_{gzx}\right]}^{T}$ is the installation angle error between the g-frame and s-frame. $S_{a}={\left[S_{ayz},S_{azy},S_{azx}\right]}^{T}$ is the installation angle error between the a-frame and s-frame. (•⊕) is the operation that constructs $S_{g}$ into $C_{g}^{s}$ . (•⊙) is the operation that constructs $S_{a}$ into $C_{a}^{s}$ .

Through the above analysis, it can be confirmed that the gyro and accelerometer errors in the r-frame are expressed as:

(15) $$\begin{array}{l} \delta \omega_{ir}^{r}=\left[\left(S_{g}⊕\right)+K_{g}+\left(\mu \times \right)\right]\omega_{ir}^{r}+\varepsilon +w_{g}\\ \delta f_{ir}^{r}=\left[\left(S_{a}⊙\right)+K_{a}+\left(\mu \times \right)\right]f_{ir}^{r}+\nabla +w_{a} \end{array}$$

where $\varepsilon ,\nabla ,K_{g},K_{a},S_{g},S_{a},\mu$ are random constant variables, that is, their derivatives are all zero. $w_{g}$ is the measurement noise of the gyros. $w_{a}$ is the measurement noise of the accelerometer.

### 3.3. Navigation Error State Model in the Inertial Frame

According to the SINS mechanization in the in0-frame in [24], the transformed dual-axis RINS mechanization in the in0-frame is provided by:

(16) $$\left[\begin{array}{c} {\dot{C}}_{r}^{in0}\\ {\dot{\bar{v}}}_{en}^{in0}\\ {\dot{r}}_{en}^{in0} \end{array}\right]=\left[\begin{array}{c} C_{r}^{in0}\left(\omega_{ir}^{r}\times \right)\\ C_{r}^{in0}f_{ir}^{r}+C_{n}^{in0}G^{n}\\ {\bar{v}}_{en}^{in0} \end{array}\right]$$

where $C_{r}^{in0}$ is the direction of the cosine matrices from the r-frame to the in0-frame. ${\bar{v}}_{en}^{in0}$ is the auxiliary velocity vector. $r_{en}^{in0}$ is the position in the in0-frame. $\omega_{ir}^{r}$ is the angular velocity expressed in the r-frame measured with the gyros. $f_{ir}^{r}$ is the specific force in the r-frame measured with the accelerometers. $G^{n}$ is the global gravitational vector, and the auxiliary velocity vector is provided by:

(17) $${\bar{v}}_{en}^{in0}=v_{en}^{in0}+\left(\omega_{in0e}^{in0}\times \right)r_{en}^{in0}$$

where $v_{en}^{in0}$ is the velocity in the in0-frame. $\omega_{in0e}^{in0}$ is the earth’s rotation in the in0-frame.

The relationship between $r_{en}^{in0}$ and geographical $p_{en}^{n}={\left[\lambda ,L,h\right]}^{T}$ is provided by:

(18) $$r_{en}^{in0}=C_{e}^{in0}r_{en}^{e}=C_{e}^{in0}\Gamma \left(p_{en}^{n}\right)$$

where λ , L , and h are the longitude, latitude, and height, respectively, obtained from the GPS.

(19) $$r_{en}^{e}=\Gamma \left(p_{en}^{n}\right)=\left[\begin{array}{c} \left(R_{N}+h\right)\cos L\cos\lambda \\ \left(R_{N}+h\right)\cos L\sin\lambda \\ \left[R_{N}\left(1-e^{2}\right)+h\right]\sin L \end{array}\right]$$

The attitude transformation matrix $C_{e}^{in0}$ is provided by:

(20) $$C_{e}^{in0}=C_{e0}^{in0}C_{e}^{e0}$$

where $C_{e0}^{in0}$ is determined by the initial location and $p_{en}^{n}\left(0\right)$ , $C_{e}^{e0}$ is determined by the earth rotation and calibration time.

(21) $$C_{e0}^{e}=\left[\begin{array}{ccc} \cos\left(\omega_{ie}t\right) & \sin\left(\omega_{ie}t\right) & 0\\ -\sin\left(\omega_{ie}t\right) & \cos\left(\omega_{ie}t\right) & 0\\ 0 & 0 & 1 \end{array}\right]C_{e0}^{in0}=\left[\begin{array}{ccc} -\sin\lambda_{0} & \cos\lambda_{0} & 0\\ -\sin L_{0}\cos\lambda_{0} & -\sin L_{0}\sin\lambda_{0} & \cos L_{0}\\ \cos L_{0}\cos\lambda_{0} & \cos L_{0}\sin\lambda_{0} & \sin L_{0} \end{array}\right]$$

The relationship between ${\bar{v}}_{en}^{in0}$ and ground velocity $v_{en}^{n}$

(22) $${\bar{v}}_{en}^{in0}=C_{e}^{in0}\left(\omega_{in0e}^{e}\times \right)r_{en}^{e}+C_{n}^{in0}v_{en}^{n}$$

It can be used to initialize ${\bar{v}}_{en}^{in0}$ for the velocity calculation in (22) and can also be used as the measurement for the dual-axis RINS calibration. $v_{en}^{n}$ can be obtained using a speed sensor, such as an odometer.

Formulating $C_{r}^{in0}$ , ${\bar{v}}_{en}^{in0}$ , and $r_{en}^{in0}$ as elements of the matrix Lie group $SE_{2}\left(3\right)$ :

(23) $$\chi =\left[\frac{C_{r}^{in0}}{0_{2\times 3}}|\frac{{\bar{v}}_{en}^{in0}r_{en}^{in0}}{I_{2\times 2}}\right]$$

According to Equation (23), the differential equation of the χ can be calculated as:

(24) $$\begin{array}{l} \dot{\chi }=\Gamma_{u}\left(\chi \right)=\chi W_{1}+W_{2}\chi \\ =\left[\begin{array}{ccc} 0 & G^{in0} & 0\\ 0 & 0 & 1\\ 0 & 0 & 0 \end{array}\right]\chi +\chi \left[\begin{array}{ccc} \left(\omega_{ir}^{r}\times \right) & f_{ir}^{r} & 0\\ 0 & 0 & -1\\ 0 & 0 & 0 \end{array}\right] \end{array}$$

It is obvious to verify that any solution $\chi_{1},\chi_{2}$ of Equation (23) satisfies Equation (24). Hence, the dynamical equation $\Gamma_{u}\left(\chi \right)$ is group affine. The group-affine system owns the log-linear property of the corresponding error propagation [23]. Such striking property is just the fundamental of the linear KF based an initial alignment with an arbitrary large misalignment [24].

(25) $$\Gamma_{u}\left(\chi_{1}\right)\chi_{2}+\chi_{1}\Gamma_{u}\left(\chi_{2}\right)-\chi_{1}\Gamma_{u}\left(I_{5}\right)\chi_{2}=\Gamma_{u}\left(\chi_{1}\chi_{2}\right)$$

If χ represents the ground truth value and $\tilde{\chi }$ represents the estimated value of χ , the invariant error δχ is defined in Table 2. In error modeling based on SO(3) , only the attitude is established in the Lie group space, and the velocity error and position error are established in the Euclidean space, which is $\delta \chi \in SO\left(3\right)+R^{6}$ . Different from the error modeling based on SO(3) , the invariant error considers the direction errors of velocity and position and mathematically solves the problem of inconsistent coordinate systems defined by velocity error and position error, which is $\delta \chi \in SE_{2}\left(3\right)$ . 

#### 3.3.1. Error State Model Based on SO(3)

Derive both sides of Equation (27) simultaneously and substitute it into Equation (16). It can be deduced that the error model in the inertial frame with $\varphi^{in0}$ , $\delta {\bar{v}}_{en}^{in0}$ , and $\delta r_{en}^{in0}$ as navigation parameter errors is:

(30) $$\begin{array}{l} {\dot{\varphi }}^{in0}=-{\tilde{C}}_{r}^{in0}\delta \omega_{ir}^{r}\\ \delta {\dot{\bar{v}}}_{en}^{in0}=\left({\tilde{C}}_{r}^{in0}{\tilde{f}}_{ir}^{r}\right)\times \varphi^{in0}+{\tilde{C}}_{r}^{in0}\delta f_{ir}^{r}\\ \delta {\dot{r}}_{en}^{in0}=\delta {\bar{v}}_{en}^{in0} \end{array}$$

Derive both sides of Equation (29) simultaneously and substitute it into Equation (16). It can be deduced that the error model in the r-frame with $\varphi^{r}$ , $\delta {\bar{v}}_{en}^{in0}$ , and $\delta r_{en}^{in0}$ as navigation parameter errors is:

(31) $$\begin{array}{l} {\dot{\varphi }}^{r}=-\left({\tilde{\omega }}_{ir}^{r}\times \right)\varphi^{r}-\delta \omega_{ir}^{r}\\ \delta {\dot{\bar{v}}}_{en}^{in0}={\tilde{C}}_{r}^{in0}\left({\tilde{f}}_{ir}^{r}\times \right)\varphi^{r}+{\tilde{C}}_{r}^{in0}\delta f_{ir}^{r}\\ \delta {\dot{r}}_{en}^{in0}=\delta {\bar{v}}_{en}^{in0} \end{array}$$

It can be seen from Equations (30) and (31) that the error state model contains ${\tilde{C}}_{r}^{in0}$ , which is dependent on the trajectory and is affected by the application environment of the calibration model.

For the above two error models, their measurement models are consistent and can be expressed as:

(32) $$z_{so}=\left[\begin{array}{c} z_{sov}\\ z_{sor} \end{array}\right]=\left[\begin{array}{c} \delta {\bar{v}}_{en}^{in0}\\ \delta r_{en}^{in0} \end{array}\right]=\left[\begin{array}{c} {\tilde{\bar{v}}}_{en}^{in0}-{\bar{v}}_{en}^{in0}\\ {\tilde{r}}_{en}^{in0}-r_{en}^{in0} \end{array}\right]+V_{so}$$

where ${\tilde{\bar{v}}}_{en}^{in0}$ and ${\tilde{r}}_{en}^{in0}$ are the velocity and position solved by the dual-axis RINS. ${\tilde{\bar{v}}}_{en}^{in0}$ and ${\tilde{r}}_{en}^{in0}$ are the velocity and position solved using Equations (18) and (22).

For the error modeling in Equations (31) and (32), the feedback correction for the navigation parameters is provided by Equation (33).

(33) $$\{\begin{array}{c} {\hat{C}}_{r}^{in0}=\exp\left({\hat{\varphi }}^{in0}\right){\tilde{C}}_{r}^{in0},{\hat{C}}_{r}^{in0}={\tilde{C}}_{r}^{in0}\exp\left({\hat{\varphi }}^{r}\right)\\ {\hat{\bar{v}}}_{en}^{in0}={\tilde{\bar{v}}}_{en}^{in0}-\delta {\bar{v}}_{en}^{in0}\\ {\hat{r}}_{en}^{in0}={\tilde{r}}_{en}^{in0}-\delta r_{en}^{in0} \end{array}$$

#### 3.3.2. Right-Invariant Error State Model Based on $SE_{2}\left(3\right)$

Denote the Lie algebra $d{\bar{x}}_{R}={\left[\varphi^{in0T},d{\bar{v}}^{in0T},dr^{in0T}\right]}^{T}$ corresponding to the invariant error $\delta \chi_{Rse}$ . According to Equation (5), we have:

(34) $$C_{r}^{in0}{\tilde{C}}_{in0}^{r}=\exp\left(\varphi^{in0}\right)\approx I_{3\times 3}+\left(\varphi^{in0}\times \right)$$

(35) $$d{\bar{v}}^{in0}=J_{l}^{-1}\left(\varphi^{in0}\right)\left({\bar{v}}_{en}^{in0}-\exp\left(\varphi^{in0}\times \right){\tilde{\bar{v}}}_{en}^{in0}\right)=-\delta {\bar{v}}_{en}^{in0}+{\tilde{\bar{v}}}_{en}^{in0}\times \varphi^{in0}$$

(36) $$dr^{in0}=J_{l}^{-1}\left(\varphi^{in0}\right)\left(r_{en}^{in0}-\exp\left(\varphi^{in0}\times \right){\tilde{r}}_{en}^{in0}\right)=-\delta r_{en}^{in0}+{\tilde{r}}_{en}^{in0}\times \varphi^{in0}$$

The differential equation of $\varphi^{in0}$ , $d{\bar{v}}^{in0}$ , and $d{\bar{r}}^{in0}$ are provided by:

(37) $$\left({\dot{\varphi }}^{in0}\times \right)={\dot{C}}_{r}^{in0}{\tilde{C}}_{in0}^{r}+C_{r}^{in0}{\dot{\tilde{C}}}_{in0}^{r}=\left(-C_{r}^{in0}\delta \omega_{ir}^{r}\right)\times$$

(38) $$d{\dot{\bar{v}}}^{in0}=-\delta {\dot{\bar{v}}}_{en}^{in0}+{\dot{\tilde{\bar{v}}}}_{en}^{in0}\times \varphi^{in0}+{\tilde{\bar{v}}}_{en}^{in0}\times {\dot{\varphi }}^{in0}=G^{in0}\times \varphi^{in0}-C_{r}^{in0}\delta f_{ir}^{r}-\left({\tilde{\bar{v}}}_{en}^{in0}\times \right)C_{r}^{in0}\delta \omega_{ir}^{r}$$

(39) $$d{\dot{r}}^{in0}=-\delta {\dot{r}}_{en}^{in0}+{\dot{\tilde{r}}}_{en}^{in0}\times \varphi^{in0}+{\tilde{r}}_{en}^{in0}\times {\dot{\varphi }}^{in0}=d{\bar{v}}^{in0}-\left({\tilde{r}}_{en}^{in0}\times \right)C_{r}^{in0}\delta \omega_{ir}^{r}$$

Add the calibration errors to the state vector. The corresponding state-space model is provided by:

(40) $$\left[\begin{array}{c} {\dot{\varphi }}^{in0}\\ d{\dot{\bar{v}}}^{in0}\\ d{\dot{r}}^{in0}\\ {\dot{x}}_{c} \end{array}\right]=\left[\begin{array}{c} \underline{\begin{array}{cc} \begin{array}{ccc} 0_{3\times 3} & 0_{3\times 3} & 0_{3\times 3}\\ \left(G^{in0}\times \right) & 0_{3\times 3} & 0_{3\times 3}\\ 0_{3\times 3} & I_{3\times 3} & 0_{3\times 3} \end{array}| & F_{Rc} \end{array}}\\ 0_{24\times 33} \end{array}\right]\left[\begin{array}{c} \varphi^{in0}\\ d{\bar{v}}^{in0}\\ dr^{in0}\\ x_{c} \end{array}\right]+\left[\begin{array}{c} \underline{\begin{array}{cc} -C_{r}^{in0} & 0_{3\times 3}\\ -\left({\tilde{\bar{v}}}_{en}^{in0}\times \right)C_{r}^{in0} & -C_{r}^{in0}\\ -\left({\tilde{r}}_{en}^{in0}\times \right)C_{r}^{in0} & 0_{3\times 3} \end{array}}\\ 0_{24\times 6} \end{array}\right]\left[\begin{array}{c} w_{g}\\ w_{a} \end{array}\right]$$

where $x_{c}={\left[\varepsilon^{T},\nabla^{T},{K_{g}}^{T},{K_{a}}^{T},{S_{g}}^{T},{S_{a}}^{T},\mu^{T}\right]}^{T}$ is the state vector of the parameters to be calibrated. $F_{Rc}$ is provided by:

(41) $$F_{Rc}=\left[\begin{array}{ccccccc} -C_{r}^{in0} & 0_{3\times 3} & -C_{r}^{in0}D\left(\omega_{ir}^{r}\right) & 0_{3\times 3} & -C_{r}^{in0}\Gamma_{g}\left(\omega_{ir}^{r}\right) & 0_{3\times 3} & C_{r}^{in0}\left(\omega_{ir}^{r}\times \right)\\ -\left({\tilde{\bar{v}}}_{en}^{in0}\times \right)C_{r}^{in0} & -C_{r}^{in0} & -\left({\tilde{\bar{v}}}_{en}^{in0}\times \right)C_{r}^{in0}D\left(\omega_{ir}^{r}\right) & -C_{r}^{in0}D\left(f_{ir}^{r}\right) & -\left({\tilde{\bar{v}}}_{en}^{in0}\times \right)C_{r}^{in0}\Gamma_{g}\left(\omega_{ir}^{r}\right) & -C_{r}^{in0}\Gamma_{a}\left(\omega_{ir}^{r}\right) & \left({\tilde{\bar{v}}}_{en}^{in0}\times \right)C_{r}^{in0}\left(\omega_{ir}^{r}\times \right)+C_{r}^{in0}\left(f_{ir}^{r}\times \right)\\ -\left({\tilde{r}}_{en}^{in0}\times \right)C_{r}^{in0} & 0_{3\times 3} & -\left({\tilde{r}}_{en}^{in0}\times \right)C_{r}^{in0}D\left(\omega_{ir}^{r}\right) & 0_{3\times 3} & -\left({\tilde{r}}_{en}^{in0}\times \right)C_{r}^{in0}\Gamma_{g}\left(\omega_{ir}^{r}\right) & 0_{3\times 3} & \left({\tilde{r}}_{en}^{in0}\times \right)C_{r}^{in0}\left(\omega_{ir}^{r}\times \right) \end{array}\right]$$

where D(•) , $\Gamma_{g}\left(•\right)$ and $\Gamma_{a}\left(•\right)$ are provided by:

(42) $$D\left(a\right)=\left[\begin{array}{ccc} a_{x} & 0 & 0\\ 0 & a_{y} & 0\\ 0 & 0 & a_{z} \end{array}\right],\Gamma_{g}\left(a\right)=\left[\begin{array}{cccccc} a_{y} & a_{z} & 0 & 0 & 0 & 0\\ 0 & 0 & a_{x} & a_{z} & 0 & 0\\ 0 & 0 & 0 & 0 & a_{x} & a_{y} \end{array}\right],\Gamma_{a}\left(a\right)=\left[\begin{array}{ccc} 0 & 0 & 0\\ a_{x} & 0 & 0\\ 0 & a_{x} & a_{y} \end{array}\right],a=\left[\begin{array}{c} a_{x}\\ a_{y}\\ a_{z} \end{array}\right]$$

The right-invariant error measurement equation is provided by:

(43) $$z_{Rse}=\left[\begin{array}{c} z_{Rsev}\\ z_{Rser} \end{array}\right]=\left[\begin{array}{c} \delta {\bar{v}}_{en}^{in0}\\ \delta r_{en}^{in0} \end{array}\right]=\left[\begin{array}{c} -d{\bar{v}}^{in0}+{\tilde{\bar{v}}}_{en}^{in0}\times \varphi^{in0}\\ -dr^{in0}+{\tilde{r}}_{en}^{in0}\times \varphi^{in0} \end{array}\right]+V_{Rse}$$

It can be seen from Equation (40) that the navigation parameter system equations in the right-invariant model are only related to the known quantity $G^{in0}$ and is trajectory-independent. Although the system is group affine and can use the linear-KF to solve non-linear problems, the calibration parameters are strongly coupled with the navigation parameters, which affects the calibration accuracy.

For the right-invariant error modeling in Equation (40), the feedback correction for the navigation parameters is provided by:

(44) $$\begin{array}{l} {\hat{C}}_{r}^{in0}=\exp\left({\hat{\varphi }}^{in0}\right){\tilde{C}}_{r}^{in0}\\ {\hat{\bar{v}}}_{en}^{in0}=\exp\left({\hat{\varphi }}^{in0}\right){\tilde{\bar{v}}}_{en}^{in0}+J_{l}\left({\hat{\varphi }}^{in0}\right)d{\hat{v}}^{in0}\\ {\hat{r}}_{en}^{in0}=\exp\left({\hat{\varphi }}^{in0}\right){\tilde{r}}_{en}^{in0}+J_{l}\left({\hat{\varphi }}^{in0}\right)d{\hat{r}}^{in0} \end{array}$$

#### 3.3.3. Left-Invariant Error State Model Based on $SE_{2}\left(3\right)$

Denote the Lie algebra $d{\bar{x}}_{L}={\left[\varphi^{rT},d{\bar{v}}^{rT},dr^{rT}\right]}^{T}$ corresponding to the invariant error $\delta \chi_{Lse}$ . According to Equation (5), we have:

(45) $${\tilde{C}}_{in0}^{r}C_{r}^{in0}=\exp\left(\varphi^{r}\right)=I_{3\times 3}+\left(\varphi^{r}\times \right)$$

(46) $$d{\bar{v}}^{r}=J_{l}^{-1}\left(\varphi^{r}\right)\left(-{\tilde{C}}_{r}^{in0T}\delta {\bar{v}}_{en}^{in0}\right)=-{\tilde{C}}_{r}^{in0T}\delta {\bar{v}}_{en}^{in0}$$

(47) $$d{\bar{r}}^{r}=J_{l}^{-1}\left(\varphi^{in0}\right)\left({\tilde{C}}_{r}^{in0T}\delta r_{en}^{in0}\right)=-{\tilde{C}}_{r}^{in0T}\delta r_{en}^{in0}$$

The differential equations of $\varphi^{r}$ , $d{\bar{v}}^{r}$ , and $d{\bar{r}}^{r}$ are provided by:

(48) $$\left({\dot{\varphi }}^{r}\times \right)={\dot{\tilde{C}}}_{in0}^{r}C_{r}^{in0}+{\tilde{C}}_{in0}^{r}{\dot{C}}_{r}^{in0}=-\left[\left(\omega_{ir}^{r}\times \right)\varphi^{r}\right]\times -\left(\delta \omega_{ir}^{r}\times \right)$$

(49) $$d{\dot{\bar{v}}}^{r}=-{\dot{\tilde{C}}}_{r}^{in0T}\delta {\bar{v}}_{en}^{in0}-{\tilde{C}}_{r}^{in0T}\delta {\dot{\bar{v}}}_{en}^{in0}=-\left({\tilde{\omega }}_{in0r}^{r}\times \right)d{\bar{v}}^{r}-\left(f_{ir}^{r}\times \right)\varphi^{r}-\delta f_{ir}^{r}$$

(50) $$d{\dot{\bar{r}}}^{r}=-{\dot{\tilde{C}}}_{r}^{in0T}\delta r_{en}^{in0}-{\tilde{C}}_{r}^{in0T}\delta {\dot{r}}_{en}^{in0}=-\left({\tilde{\omega }}_{in0r}^{r}\times \right)d{\bar{r}}^{r}+d{\bar{v}}^{r}$$

Add the calibration errors to the state vector. The corresponding state-space model is provided by:

(51) $$\left[\begin{array}{c} {\dot{\varphi }}^{r}\\ d{\dot{\bar{v}}}^{r}\\ d{\dot{r}}^{r}\\ {\dot{x}}_{c} \end{array}\right]=\left[\begin{array}{c} \underline{\begin{array}{cc} \begin{array}{ccc} -\left({\tilde{\omega }}_{ir}^{r}\times \right) & 0_{3\times 3} & 0_{3\times 3}\\ -\left({\tilde{f}}_{ir}^{r}\times \right) & -\left({\tilde{\omega }}_{ir}^{r}\times \right) & 0_{3\times 3}\\ 0_{3\times 3} & I_{3\times 3} & -\left({\tilde{\omega }}_{ir}^{r}\times \right) \end{array}| & \begin{array}{c} F_{Lc}\\ 0_{3\times 24} \end{array} \end{array}}\\ 0_{24\times 33} \end{array}\right]\left[\begin{array}{c} \varphi^{r}\\ d{\bar{v}}^{r}\\ dr^{r}\\ x_{c} \end{array}\right]+\left[\begin{array}{c} \underline{\begin{array}{cc} -I_{3\times 3} & 0_{3\times 3}\\ 0_{3\times 3} & -I_{3\times 3}\\ 0_{3\times 3} & 0_{3\times 3} \end{array}}\\ 0_{24\times 6} \end{array}\right]\left[\begin{array}{c} w_{g}\\ w_{a} \end{array}\right]$$

where $F_{Lc}$ is provided by:

(52) $$F_{Lc}=\left[\begin{array}{ccccccc} -I_{3\times 3} & 0_{3\times 3} & -D\left(\omega_{ir}^{r}\right) & 0_{3\times 3} & -\Gamma_{g}\left(\omega_{ir}^{r}\right) & 0_{3\times 3} & \left(\omega_{ir}^{r}\times \right)\\ 0_{3\times 3} & -I_{3\times 3} & 0_{3\times 3} & -D\left(f_{ir}^{r}\right) & 0_{3\times 6} & -\Gamma_{a}\left(\omega_{ir}^{r}\right) & \left(f_{ir}^{r}\times \right) \end{array}\right]$$

The left-invariant error measurement equation is provided by:

(53) $$z_{Lse}=\left[\begin{array}{c} z_{Lsev}\\ z_{Lser} \end{array}\right]=\left[\begin{array}{c} \delta {\bar{v}}_{en}^{in0}\\ \delta r_{en}^{in0} \end{array}\right]=\left[\begin{array}{c} -{\tilde{C}}_{r}^{in0}dv^{r}\\ -{\tilde{C}}_{r}^{in0}dr^{r} \end{array}\right]+V_{Lse}$$

Compared with the right-invariant model, ${\tilde{C}}_{r}^{in0}$ is only in the measurement equation of the left-invariant model, and the system equation is only related to $\omega_{ir}^{r}$ and $f_{ir}^{r}$ , which is less dependent on the trajectory and its error propagation would be autonomous.

For the left-invariant error modeling in Equation (51), the feedback correction for the navigation parameters is provided by:

(54) $$\begin{array}{l} {\hat{C}}_{r}^{in0}={\tilde{C}}_{r}^{in0}\exp\left({\hat{\varphi }}^{r}\right)\\ {\hat{\bar{v}}}_{en}^{in0}={\tilde{\bar{v}}}_{en}^{in0}+{\tilde{C}}_{r}^{in0}J_{l}\left({\hat{\varphi }}^{r}\right)d{\hat{v}}^{r}\\ {\hat{r}}_{en}^{in0}={\tilde{r}}_{en}^{in0}+{\tilde{C}}_{r}^{in0}J_{l}\left({\hat{\varphi }}^{r}\right)d{\hat{r}}^{r} \end{array}$$

In summary, the left-invariant error model is selected as the filtering algorithm in this paper. The state variables of the proposed method are chosen as follows:

(55) $$X={\left[\varphi^{rT},d{\bar{v}}^{rT},\varepsilon^{T},\nabla^{T},{K_{g}}^{T},{K_{a}}^{T},{S_{g}}^{T},{S_{a}}^{T},\mu^{T}\right]}^{T}$$

where $\varphi^{r}=\left[\varphi_{x}^{r},\varphi_{y}^{r},\varphi_{z}^{r}\right]$ is left-invariant attitude error in the Euler angles. $d{\bar{v}}^{r}=\left[dv_{x}^{r},dv_{y}^{r},dv_{z}^{r}\right]$ is left-invariant velocity error. $\varepsilon =\left[\varepsilon_{x},\varepsilon_{y},\varepsilon_{z}\right]$ is the gyro drift error. $\nabla =\left[\nabla_{x},\nabla_{y},\nabla_{z}\right]$ is the accelerometer bias error. $K_{g}=\left[K_{gx},K_{gy},K_{gz}\right]$ is the gyro-scale factor error. $K_{a}=\left[K_{ax},K_{ay},K_{az}\right]$ is the accelerometer-scale factor error. $S_{a}=\left[S_{ayx},S_{azx},S_{azy}\right]$ is the accelerometer installation angle error. $S_{g}=\left[S_{gxy},S_{gxz},S_{gyx},S_{gyz},S_{gzx},S_{gzy}\right]$ is the gyro installation angle error. $\mu =\left[\mu_{x},\mu_{y},\mu_{z}\right]$ is installation angle error between the s-frame and r-frame.

The state equation and the measurement equation can be designed as follows:

(56) $$\begin{array}{l} \dot{X}=FX+GW\\ Z_{Lse}=\delta {\bar{v}}_{en}^{in0}=H_{Lse}X+V_{Lse} \end{array}$$

where W and $V_{Lse}$ are the process noise and measurement noise, respectively. G is the process noise matrix. The matrix F and the matrix $H_{Lse}$ are expressed as:

(57) $$F=\left[\begin{array}{c} \underline{\begin{array}{cc} \begin{array}{cc} -\left({\tilde{\omega }}_{ir}^{r}\times \right) & 0_{3\times 3}\\ -\left({\tilde{f}}_{ir}^{r}\times \right) & -\left({\tilde{\omega }}_{ir}^{r}\times \right) \end{array}| & F_{Lc} \end{array}}\\ 0_{24\times 30} \end{array}\right],H_{Lse}=\left[\begin{array}{ccc} 0_{3\times 3} & -{\tilde{C}}_{r}^{in0} & 0_{3\times 24} \end{array}\right]$$

The algorithm flow for discretizing the continuous Kalman filter is shown in Algorithm 1.

**Algorithm 1** Discrete Kalman filter algorithm flow(1) State and variance matrix initialization


${\hat{X}}_{0}=X\left(t_{0}\right),{\hat{P}}_{0}=P\left(t_{0}\right)$

(2) State propagation process


$\begin{array}{l} {\hat{X}}_{k/k-1}=\Phi_{k/k-1}{\hat{X}}_{k-1}\\ {\hat{P}}_{k/k-1}=\Phi_{k/k-1}{\hat{P}}_{k-1}\Phi_{k/k-1}^{T}+\Gamma_{k-1}Q\Gamma_{k-1}^{T}\\ \Phi_{k/k-1}\approx I+F\left(t_{k-1}\right)\Delta T+F\left(t_{k-1}\right)F\left(t_{k-1}\right)\Delta T^{2}/2\\ \Gamma_{k-1}\approx \left(I+F\left(t_{k-1}\right)\Delta T/2+F\left(t_{k-1}\right)F\left(t_{k-1}\right)\Delta T^{2}/6\right)G \end{array}$

(3) Update filter state


$\begin{array}{l} K_{k}=P_{k/k-1}H_{k}^{T}{\left(H_{k}P_{k/k-1}H_{k}^{T}+R_{k}\right)}^{-1}\\ P_{k}=\left(I-K_{k}H_{k}\right)P_{k/k-1}\\ {\hat{X}}_{k}={\hat{X}}_{k/k-1}+K_{k}\left(z-H_{k}{\hat{X}}_{k/k-1}\right) \end{array}$

(4) Update navigation state
where 
ΔT
 is the discrete time. 
Q
 and 
R
 are device random noise.

## 4. The Fast Self-Calibration Method and Observability Analysis of the Dual-Axis RINS

### 4.1. Rotation Scheme Design and Observability Analysis

**Figure 4:**
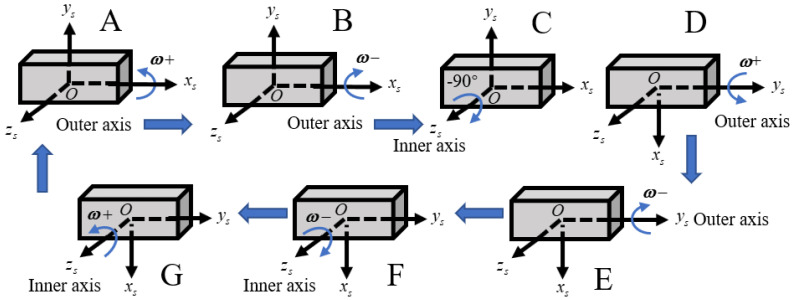
One period of rotation scheme.

In order to accurately estimate all the state variables in the self-calibration procedure, the rotation scheme should be properly designed to ensure that all the state variables are observable. Reference [35] proposed design principles for a rotation scheme, which included pointing upward and downward alternately for each accelerometer and a bidirectional rotation on each gyro. According to the above principles and the limitations of the gimbal mechanism, the rotation scheme proposed in this article is shown in Figure 4. In a rotation period, first, the outer axis rotates 270 degrees and −270 degrees (corresponding to A and B in Figure 4). Then, the inner axis rotates to −90 degrees (corresponding to C in Figure 4), and the outer axis rotates bidirectionally (corresponding to D and E in Figure 4). Finally, the inner axis rotates bidirectionally (corresponding to F and G in Figure 4) and returns to position A. During the rotation, the angular velocity is ω , and it stops for $T_{s}$ seconds after each rotation of 90 degrees. According to Equation (16), except for ε and ∇ , the remaining calibration errors require $\omega_{ir}^{r}$ and $f_{ir}^{r}$ to excite. In order to excite the error of the gyros, the gyros should be continuously sensitive to the angular velocity of the gimbal mechanism. In the same way, to excite the error of the accelerometers, the accelerometer should be in the vertical direction of the horizontal plane for a long time. Therefore, in order to fully excite the IMU error, ω is set to 18°/s and the stop time $T_{s}$ is set to 30 s. The complete rotation period is 600 s.

This article uses the PWCS method to verify the feasibility of the designed rotation scheme. In each short time period, the state matrix and measurement matrix are assumed to be constant matrices. At this time, the linear time-varying system transforms into a linear time-invariant system, and the observable matrix of each time period can be obtained. In the i-th time period, the observability matrix of the dynamic system can be expressed as:

(58) $$Q_{i}={\left[{H_{i}}^{T},{\left(H_{i}F_{i}\right)}^{T},{\left(H_{i}F_{i}^{2}\right)}^{T},\cdots ,{\left(H_{i}F_{i}^{n-1}\right)}^{T}\right]}^{T}$$

where $H_{i}$ and $F_{i}$ are the measurement matrix and the state matrix in the i-th time interval and n is the dimension of the state. The stripped observability matrix (SOM) is:

(59) $$Q_{SOM}\left(r\right)={\left[Q_{1}^{T},Q_{2}^{T},Q_{3}^{T},\cdots ,Q_{r}^{T}\right]}^{T}$$

When the rank of $Q_{SOM}\left(r\right)$ is n, the system is fully observable, while if the rank of $Q_{SOM}\left(r\right)$ is less than n, the system is not fully observable. Performing a singular value decomposition on $Q_{SOM}\left(r\right)$ , Equation (60) can be obtained:

(60) $$Q_{SOM}\left(r\right)=U\Sigma V^{T}=\sum_{i=1}^{n}u_{i}\sigma_{i}v_{i}^{T}$$

where $U=\left[u_{1},u_{2}\cdots u_{rmn}\right]$ and $V=\left[v_{1},v_{2}\cdots v_{n}\right]$ are rmn×rmn -dimensional and n×n -dimensional unitary matrices, respectively. $\Sigma ={\left[\Sigma_{0},0_{n\times (rmn-n)}\right]}^{T}$ , $\Sigma_{0}=\mathrm{diag}\left(\sigma_{1},\sigma_{2},\cdots \sigma_{n}\right)$ , and $\sigma_{i}\left(i=1,\cdots ,m\right)$ are the singular values of $Q_{SOM}\left(r\right)$ .

Define the singular value $\sigma_{k}$ corresponding to any element $X_{k}$ of the state vector X to be the singular value $\sigma_{i}$ that maximizes ${\left\{\left(u_{i}^{T}Y/\sigma_{i}\right)v_{i}\right\}}_{k}\left(i=1,2,\cdots ,m\right)$ . The observability degree of $X_{k}$ is defined as:

(61) $$\eta_{k}=\sigma_{k}/\sigma_{0}$$

where $Y={\left[Y_{1}^{T},Y_{2}^{T},\cdots Y_{r}^{T}\right]}^{T}$ , $Y_{i}={\left[Z_{i}^{T},{\dot{Z}}_{i}^{T},\cdots Z^{\left(n-1\right)T}\right]}^{T}$ is the measurement in each time period, and its derivatives of each order. $\sigma_{0}$ is the singular value corresponding to the state component that can be directly obtained from external information. 

Corresponding to the left-invariant error model of Equation (57), the rank of the system is 27. $Q_{SOM}\left(r\right)$ is less than the state dimension, indicating that the system is not completely observable. Calculate the observability degrees of each variable at the end of calibration. The observable degrees of $\varepsilon_{x}$ , $\varepsilon_{y}$ , $\varepsilon_{z}$ , $\nabla_{x}$ , $\nabla_{y}$ , $\nabla_{z}$ , $k_{ax}$ , $k_{ay}$ , $k_{az}$ , $k_{gx}$ , $k_{gy}$ , $k_{gz}$ , $S_{gxy}$ , $S_{gxz}$ , $S_{gyx}$ , $S_{gyz}$ , $S_{gzx}$ , $S_{gzy}$ , $S_{ayx}$ , $S_{azx}$ , $S_{azy}$ , α , β , and γ are 2.23 × 10^−4^, 2.25 × 10^−4^, 1.48 × 10^−4^, 0.062, 0.063, 0.062, 8.56 × 10^−4^, 7.51 × 10^−5^, 2.3 × 10^−8^, 0.29, 0.33, 0.61, 5.52 × 10^−4^, 7.71 × 10^−6^, 6.31 × 10^−4^, 7.71 × 10^−4^, 5.78 × 10^−4^, 5.07 × 10^−4^, 0.21, 0.26, 0.29, 2.29 × 10^−14^, 2.29 × 10^−14^, and 3.64 × 10^−16^ respectively. The observability degrees of α , β , and γ are the worst and is unobservable, which is the reason why the rank of $Q_{SOM}\left(r\right)$ and n differ by 3. The system error of the gyros and the installation error angle between the r-frame and the s-frame are poorly observable degrees. In response to this situation, the angular velocity constraint equation is added to the measurement equation to render all the parameters observable.

### 4.2. Angular Velocity Constraint Equation

During the calibration process, the gimbal mechanism drives the IMU rotation. The angular rate of the gimbal mechanism can be utilized as a reference to calibrate the systematic errors of the gyros and the installation error between the s-frame and the r-frame. The outputs of the gyros in the s-frame can be expressed as follows:

(62) $$\omega_{ig}^{g}=C_{s}^{g}C_{r}^{s}\left(C_{in0}^{r}\omega_{ie}^{in0}+\omega_{br}^{r}+\varepsilon \right)+w_{g}$$

where $\omega_{ie}^{in0}={\left[0,\omega_{ie}\cos L_{0},\omega_{ie}\sin L_{0}\right]}^{T}$ is the angular velocity of the earth expressed in the in0-frame. $L_{0}$ is the latitude in the initial location. $\omega_{br}^{r}$ is the angular velocity of the gimbal mechanism.

By substituting Equations (11) and (12) into Equation (62), the second-order small quantity can be ignored and the IMU measurement constraint equation can be expressed as:

(63) $$\begin{array}{l} \omega_{ig}^{g}=\left(I_{3\times 3}+S_{g}⊕+K_{g}+\mu \times \right)\omega_{1}+\varepsilon +w_{g}\\ \omega_{1}=C_{in0}^{r}\omega_{ie}^{in0}+\omega_{br}^{r} \end{array}$$

Equation (63) can be converted into the measurement equation of the calibration parameters:

(64) $$\begin{array}{l} Z_{AVC}=H_{AVC}X+V_{AVC}=\omega_{ig}^{g}-\omega_{1}\\ H_{AVC}=\left[0_{3\times 6},I_{3\times 3},0_{3\times 3},D\left(\omega_{1}\right),0_{3\times 3},\Gamma_{g}\left(\omega_{1}\right),0_{3\times 3},\left(\omega_{1}\right)\times \right] \end{array}$$

Combine Equations (32), (57) and (64) to obtain the new extended measurement equation:

(65) $$Z_{LseAVC}=H_{LseAVC}X+V_{LseAVC}={\left[Z_{Lse}^{T},Z_{AVC}^{T}\right]}^{T},H={\left[H_{Lse}^{T},H_{AVC}^{T}\right]}^{T}$$

(66) $$Z_{soAVC}=H_{soAVC}X+V_{soAVC}={\left[z_{zov}^{T},Z_{AVC}^{T}\right]}^{T},H_{soAVC}={\left[H_{sov}^{T},H_{AVC}^{T}\right]}^{T}$$

where $H_{sov}=\left[0_{3\times 3},I_{3\times 3},0_{3\times 24}\right]$ .

For the left-invariant error model with F and H as the system matrix and the measurement matrix, its rank of $Q_{SOM}\left(r\right)$ is 30. The observability degrees of $\varepsilon_{x}$ , $\varepsilon_{y}$ , $\varepsilon_{z}$ , $\nabla_{x}$ , $\nabla_{y}$ , $\nabla_{z}$ , $k_{ax}$ , $k_{ay}$ , $k_{az}$ , $k_{gx}$ , $k_{gy}$ , $k_{gz}$ , $S_{gxy}$ , $S_{gxz}$ , $S_{gyx}$ , $S_{gyz}$ , $S_{gzx}$ , $S_{gzy}$ , $S_{ayx}$ , $S_{azx}$ , $S_{azy}$ , α , β , and γ are 0.98, 0.98, 0.98, 2.71 × 10^−4^, 2.72 × 10^−4^, 2.71 × 10^−4^, 0.2, 0.2, 0.2, 2.41 × 10^−3^, 2.21 × 10^−3^, 2.36× 10^−3^, 0.23, 0.21, 0.21, 0.21, 0.22, 0.21, 1.3 × 10^−3^, 1.1 × 10^−3^, 8.9 × 10^−4^, 0.38, 0.35, and 0.23, respectively. The angular velocity constraint equation is added to the measurement equations, which improves the system’s observability, and all calibration parameters can be observed. 

From the perspective of the analytical analysis of the variable observability, the accelerometer calibration error has a linear relationship with the first derivative of the velocity error. The gyro error is linearly related to the derivative of the second-order velocity error. It can be seen from the angular velocity constraint equation that there is a linear relationship between the gyros systematic error parameter and $Z_{g}$ . Adding it to the measurement equation can more directly observe the gyro system error and the installation error between the s-frame and r-frame, effectively shortening the calibration time.

### 4.3. Backtracking Scheme Design in the Inertial Frame

**Figure 5:**
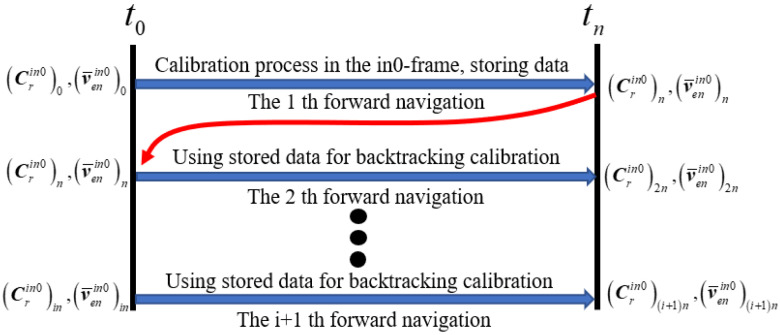
Backtracking scheme.

Backtracking navigation reduces the calibration time by utilizing stored data multiple times. The backtracking scheme is shown in Figure 5. During the calibration process, the SINS update algorithm in the inertial frame is applied for forward navigation. During the first forward navigation process, the data of the IMU and gimbal mechanism are stored. Subsequently, using the stored data multiple times, the calibration parameter error can be accurately estimated in a short time.

## 5. Simulation Test and Experimental Analysis

This section is devoted to numerically evaluating the calibration performance based on different error state models. The calibration method based on the error state model (30) and measurement model (32) is denoted as RSO-KF. The calibration method based on the error state model (31) and measurement model (32) is denoted as LSO-KF. The calibration method based on the error state model (30) and measurement model (65) is denoted as RSOAVC-KF. The calibration method based on the error state model (31) and measurement model (65) is denoted as LSOAVC-KF. The calibration method based on the error state model (40) and measurement model (66) is denoted as RSEAVC-KF. The calibration method based on the error state model (51) and measurement model (66) is denoted as LSEAVC-KF (the proposed method). The above methods all apply the backtracking method to unify the algorithm process and verify the impact of different error modeling methods on the calibration algorithm. During the simulation and experiment, the dual-axis RINS remained stationary. The location was obtained from a GPS, and the speed was zero.

### 5.1. Simulation Test

To verify the effectiveness of the proposed algorithm, the simulations are demonstrated in this section. The initial location was 31.94° N, 118.79° E, 15 m. The initial attitude error was 0.1°(roll), 0.1°(pitch), 0.5°(heading). The random gyro drifts were 0.005 deg/h, and the random accelerometer bias was 50 μg. The error of the IMU-scale factor was 100 ppm. The error of the IMU installation angle was 20″. The error of installation angle between the s-frame and r-frame was 200″. The error of the angle of the gimbal mechanism was 0.5″. The error of the angular rate of the gimbal mechanism was 0.7″/s. The rotation scheme is consistent with that described in Section 4.1. The number of backtracking navigations was four.

The simulation was divided into two parts. First, the validity of the angular velocity constraints was verified. Then, the advantages of accuracy were verified and compared with the error modeling method based on SO(3) .

#### 5.1.1. Verification of the Observability Analysis

**Figure 6:**
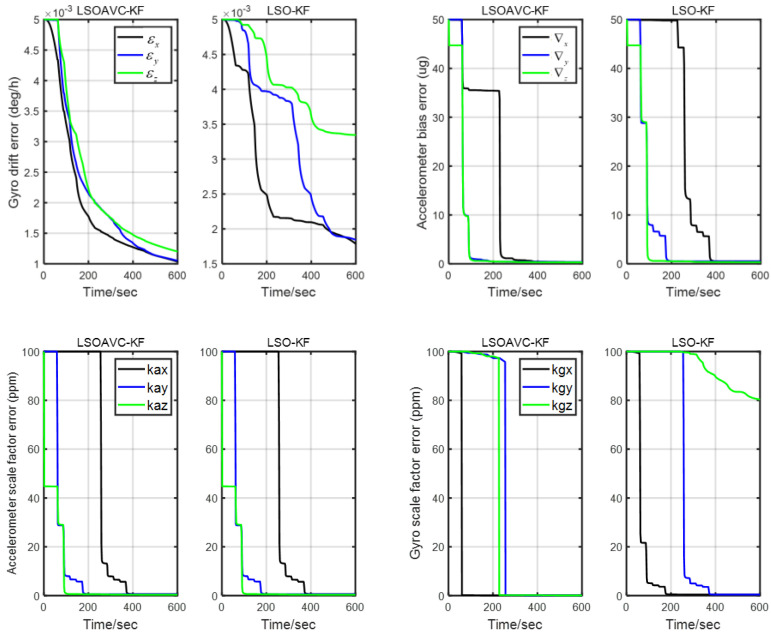

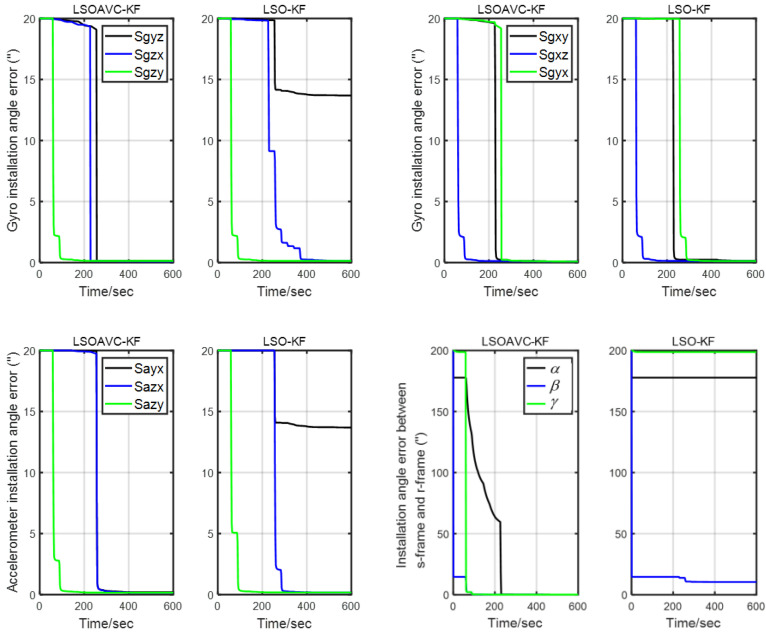
RMSs of the calibration error covariance.

The observability was assessed with a covariance analysis. The convergence process of the root mean square (RMS) of the calibration error covariance is shown in Figure 6. It can be seen from Figure 6 that the RMSs of the $k_{gz}$ , $S_{gyz}$ , $S_{ayx}$ , α , and γ error covariance are large. The observability is consistent with the analysis in Section 4. After introducing AVC constraints into the measurement equation, all the calibration error parameters could be estimated well. Moreover, the RMS of the error covariance became smaller faster.

#### 5.1.2. Comparison of the Calibration Accuracy

In order to eliminate the influence of unobservable installation errors in traditional calibration algorithms, AVC constraints are added as a comparison algorithm to verify the superiority of the calibration algorithm based on invariant error modeling. The four algorithms are evaluated for comparison.

**Figure 7:**
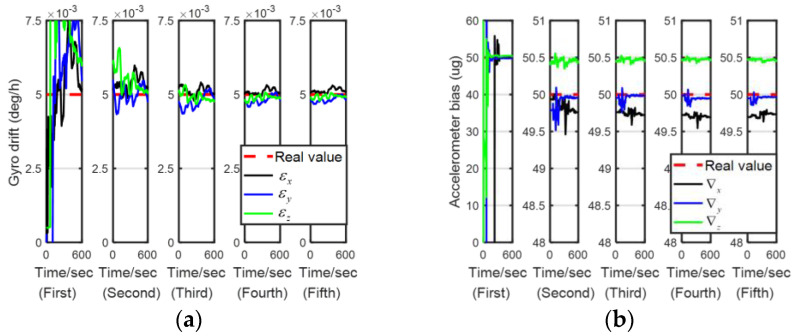
Estimation of IMU bias errors. (**a**) $\varepsilon_{x}$ , $\varepsilon_{y}$ , $\varepsilon_{z}$ ; (**b**) $\nabla_{x}$ , $\nabla_{y}$ , $\nabla_{z}$ .

**Figure 8:**
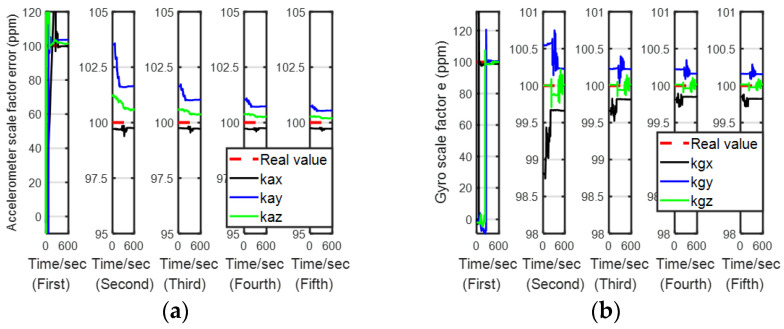
Estimation of IMU-scale factor errors. (**a**) $k_{ax}$ , $k_{ay}$ , $k_{az}$ ; (**b**) $k_{gx}$ , $k_{gy}$ , $k_{gz}$ .

**Figure 9:**
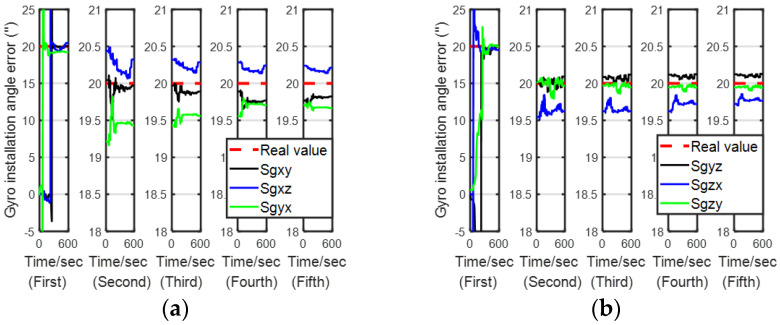
Estimation of gyro installation angle errors. (**a**) $S_{gxy}$ , $S_{gxz}$ , $S_{gyx}$ (**b**) $S_{gyz}$ , $S_{gzx}$ , $S_{gzy}$ .

**Figure 10:**
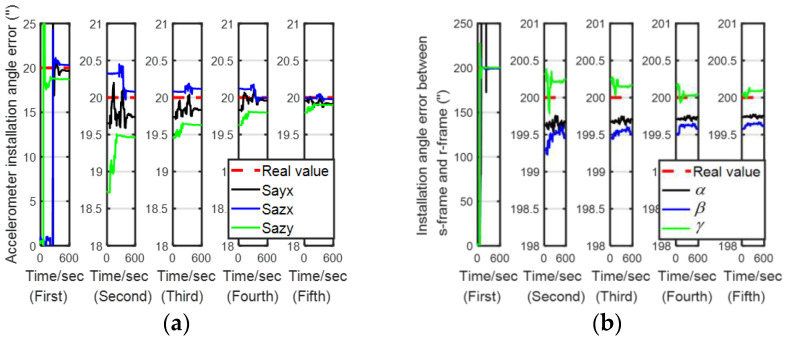
Estimation of accelerometer installation angle errors. (**a**) $S_{ayx}$ , $S_{azx}$ , $S_{azy}$ ; estimation of installation angle errors between the s-frame and r-frame. (**b**) α , β , and γ .

Figure 7, Figure 8, Figure 9 and Figure 10 describe the estimation results of the calibration errors; the red dotted lines represent real values, and the solid lines represent the estimation result of the proposed method (LSEAVC-KF). It took 600 s in the first stage, and then the time spent on backtracking navigation was calculated using the Matlab platform to be 106.1 s. We can see all errors present good convergence with the proposed method within 12 min. Except for the gyro drift error, the rest of the calibration errors converged to near the true value in the first stage. The estimation error of the gyro drift was less than 4%, and the rest of the calibration errors were less than 2%.

In order to further verify the superiority of the proposed method, the estimation accuracy of the remaining three methods was compared, and 100 Monte Carlo simulations were performed for the calibration. The statistical results of the mean error (ME) and root mean square error (RMSE) of each error parameter are shown in Table 3. Among the four methods, LSEAVC-KF had the best effect. The error of the invariant error modeling method was significantly smaller than the error of the error modeling method based on SO(3) , which proves that the invariant error can express the error characteristics more accurately.

In order to prove the calibration performance of LSEAVC-KF in the case of large misalignment angles, the calibration errors under different heading angle errors are shown in Table 4. With an increasing heading angle error, it can be observed that the estimation error experiences a slight growth, but it remains within an acceptable range. The proposed algorithm demonstrates greater applicability and can accurately calibrate errors even in harsh environmental conditions. In comparison to traditional calibration methods, it is less reliant on complex instrumentation and offers a faster convergence rate.

### 5.2. Experiment

**Figure 11:**
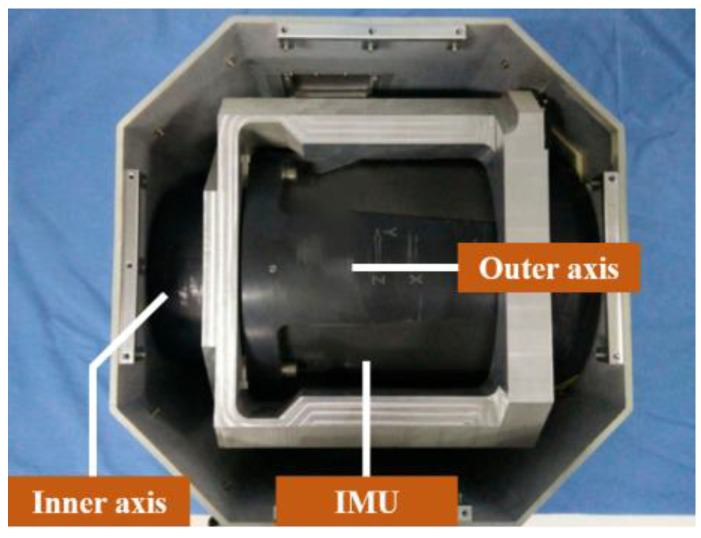
Internal structure of the dual-axis rotational inertial navigation system (RINS).

In order to verify the feasibility of the proposed method in the actual environment, this paper uses a dual-axis SINS to conduct the experiments, as shown in Figure 11. The dual-axis SINS consists of three fiber optic gyroscopes and three quartz accelerometers. The specific performance is shown in Table 5.

**Figure 12:**
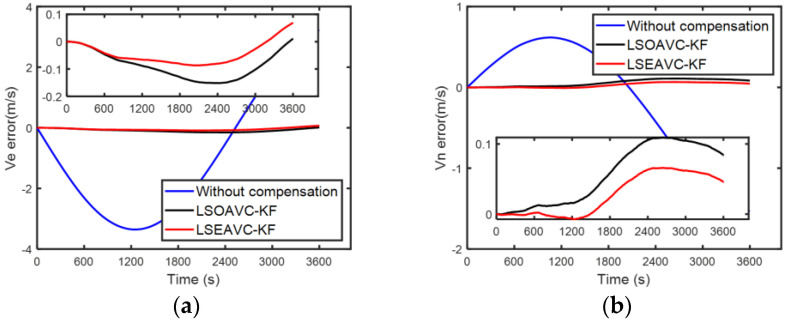
(**a**) Velocity errors along east. (**b**) Velocity errors along north.

**Figure 13:**
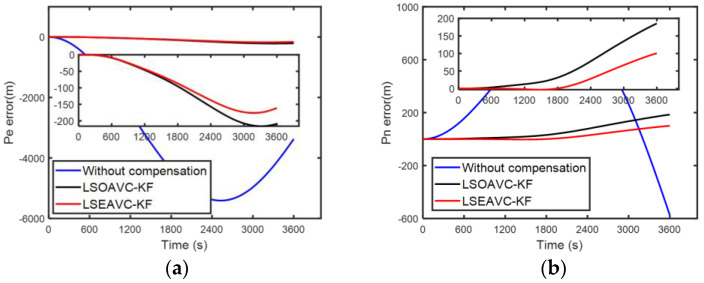
(**a**) Position errors along east. (**b**) Position errors along north.

Since the true error of the IMU cannot be known, the accuracy of the error parameter estimation was verified through navigation performance. The higher the accuracy of the IMU parameter, the better the navigation performance. Hence, a navigation experiment with different calibration results was adopted. LSOAVC-KF was chosen as the traditional algorithm for comparison. The velocity errors and position errors under different calibration results are shown in Figure 12 and Figure 13. 

The statistical results of the navigation errors are listed in Table 6. In Table 6, Ve and Vn denote the east and north velocities, respectively, in the navigation frame. Pe and Pn denote the east and north positions, respectively, in the navigation frame. It can be seen in Table 6 and in Figure 12 and Figure 13 that the navigation performance of the inertial navigation system was improved with LSEAVC-KF. The maximum error and root mean square error of LSEAVC-KF are smaller than those of LSOAVC-KF.

Therefore, it can be seen from the simulation and experimental results that the proposed algorithm can quickly and accurately estimate the IMU error and installation error between the r-frame and s-frame within 12 min, effectively improving the navigation performance of a dual-axis rotational inertial navigation system.

## 6. Conclusions

Inertial navigation systems require rapid and accurate error calibration to ensure their performance. A system-level self-calibration method for a dual-axis rotational inertial navigation system based on invariant errors is proposed. Navigation errors are represented as an element of $SE_{2}\left(3\right)$ , allowing the system to converge quickly even during large misalignment angles. Compared to error modeling methods based on SO(3) , the proposed method is not affected by the calibration environment and does not require a coarse alignment. The method utilizes the information from the gimbal mechanism to establish angular velocity constraint equations, rendering all errors simultaneously estimated. The rotation scheme for the dual-axis rotational inertial navigation system is designed and discussed, and the observability of the system is analyzed using the PWCS method and an SVD-based observability analysis method. A backtracking navigation algorithm is used to shorten the calibration time. Finally, the superiority of the proposed algorithm is verified through simulations and laboratory experiments, demonstrating that the algorithm can rapidly and accurately estimate IMU systematic errors and installation errors between the r-frame and s-frame. Therefore, the proposed calibration algorithm has significant practical value and can significantly improve navigation accuracy.

## Figures and Tables

**Table 1:** Definitions of the reference frames.

Reference Frame	Definition
a-frame	Accelerometer installation frame
g-frame	Gyro installation frame
s-frame	Sensor frame
s0-frame	Sensor frame in the initial state
b-frame	Body frame, which is the orthogonal reference frame of a dual-axis RINS. In this paper, the b-frame coincides with the s0-frame
n-frame	The navigation frame, which is the orthogonal reference frame aligned with the east-north-up geodetic axes
in0-frame	The navigation inertial frame, which is the fixed axes of the n-frame in the inertial space at the beginning of the calibration process
e-frame	Earth-centered earth-fixed (ECEF) frame
e0-frame	ECEF inertial frame in the initial state
r-frame	r-frame, which is the orthogonal reference frame of the gimbal mechanism. Its three axes are defined by $x_{r},y_{r},z_{r}$ . $x_{r}$ coincides with the outer axis, $z_{r}$ coincides with the inner axis, while $y_{r}$ forms a right-handed orthogonal frame with $x_{r}$ and $z_{r}$
r0-frame	r-frame in the initial state

**Table 2:** Definitions of invariant error.

Invariant Error Model Based on $SE_{2}\left(3\right)$	Name	Corresponds to Error Model Based on SO(3)
$\begin{array}{l} \delta \chi_{Rse}=\chi {\tilde{\chi }}^{-1}=\\ \left[\frac{C_{r}^{in0}{\tilde{C}}_{r}^{in0T}}{0_{2\times 3}}|\frac{{\bar{v}}_{en}^{in0}-C_{r}^{in0}{\tilde{C}}_{r}^{in0T}{\tilde{\bar{v}}}_{en}^{in0}r_{en}^{in0}-C_{r}^{in0}{\tilde{C}}_{r}^{in0T}{\tilde{r}}_{en}^{in0}}{I_{2\times 2}}\right] \end{array}$ (26)	Right Invariant	$\begin{array}{l} I_{3\times 3}+\left(\varphi^{in0}\times \right)=C_{r}^{in0}{\tilde{C}}_{r}^{in0T}\\ \delta {\bar{v}}_{en}^{in0}={\tilde{\bar{v}}}_{en}^{in0}-{\bar{v}}_{en}^{in0}\\ \delta r_{en}^{in0}={\tilde{r}}_{en}^{in0}-r_{en}^{in0} \end{array}$ (27)
$\begin{array}{l} \delta \chi_{Lse}={\tilde{\chi }}^{-1}\chi =\\ \left[\frac{{\tilde{C}}_{r}^{in0T}C_{r}^{in0}}{0_{2\times 3}}|\frac{{\tilde{C}}_{r}^{in0T}\left({\bar{v}}_{en}^{in0}-{\tilde{\bar{v}}}_{en}^{in0}\right){\tilde{C}}_{r}^{in0T}\left(r_{en}^{in0}-{\tilde{r}}_{en}^{in0}\right)}{I_{2\times 2}}\right] \end{array}$ (28)	Left Invariant	$\begin{array}{l} I_{3\times 3}+\left(\varphi^{r}\times \right)={\tilde{C}}_{r}^{in0T}C_{r}^{in0}\\ \delta {\bar{v}}_{en}^{in0}={\tilde{\bar{v}}}_{en}^{in0}-{\bar{v}}_{en}^{in0}\\ \delta r_{en}^{in0}={\tilde{r}}_{en}^{in0}-r_{en}^{in0} \end{array}$ (29)

**Table 3:** Simulation results from the four methods.

Parameter	RSEAVC-KF		RSOAVC-KF		LSEAVC-KF		LSOAVC-KF	
	ME	RMSE	ME	RMSE	ME	RMSE	ME	RMSE
$\varepsilon_{x}$ (°/h)	0.0003	0.0042	0.0005	0.0058	0.0002	0.0035	0.0004	0.0052
$\varepsilon_{y}$ (°/h)	0.0006	0.0041	0.0003	0.0042	0.0005	0.0039	0.0002	0.0042
$\varepsilon_{z}$ (°/h)	−0.0003	0.0034	−0.0004	0.0039	−0.0001	0.0020	−0.0005	0.0039
$\nabla_{x}$ (μg)	−0.2131	0.8136	−0.2794	0.8935	−0.1434	0.7252	−0.2327	0.868
$\nabla_{y}$ (μg)	0.1595	0.74	0.3689	0.8136	0.0796	0.7375	0.2049	0.744
$\nabla_{z}$ (μg)	0.053	0.3953	0.1245	0.4452	0.0436	0.3771	0.0983	0.44
$k_{ax}$ (ppm)	0.1447	0.1668	0.1808	0.1938	0.1429	0.1644	0.1644	0.169
$k_{ay}$ (ppm)	−0.1493	0.1658	−0.152	0.169	−0.1464	0.1646	−0.1517	0.1674
$k_{az}$ (ppm)	−0.1449	0.1584	−0.1561	0.1739	−0.1396	0.1535	−0.1553	0.1585
$k_{gx}$ (ppm)	0.0321	0.7175	−0.2059	0.7305	−0.0222	0.6592	−0.1809	0.7255
$k_{gy}$ (ppm)	0.4286	0.704	0.5241	1.911	0.3087	0.6776	0.5095	0.9855
$k_{gz}$ (ppm)	0.0267	0.3216	0.0654	0.4094	0.0055	0.298	0.061	0.389
$S_{gxy}$ (″)	0.0376	0.3052	0.1986	0.3549	0.015	0.2579	0.1828	0.3166
$S_{gxz}$ (″)	0.0594	0.2630	0.0811	0.2888	0.0523	0.2264	0.0697	0.2843
$S_{gyx}$ (″)	0.2352	0.3658	0.4452	0.5174	0.2215	0.3441	0.3935	0.51
$S_{gyz}$ (″)	0.0532	0.1663	0.0762	0.1759	0.0526	0.1375	0.07	0.1702
$S_{gzx}$ (″)	0.3509	0.4442	0.4905	0.5414	0.3338	0.4142	0.4750	0.4712
$S_{gzy}$ (″)	0.4519	0.4801	0.4741	0.4991	0.3329	0.3547	0.4523	0.4833
$S_{ayx}$ (″)	0.1472	0.3518	0.4257	0.5364	0.1296	0.3454	0.3866	0.4828
$S_{azx}$ (″)	0.3752	0.5953	0.542	0.6552	0.365	0.4648	0.5214	0.6058
$S_{azy}$ (″)	0.3949	0.4742	0.5194	0.5836	0.3363	0.4468	0.5133	0.5067
α (″)	0.1522	0.2978	0.2748	0.3139	0.1301	0.1754	0.2526	0.3022
β (″)	−0.115	0.3132	−0.2536	0.3381	−0.112	0.2757	−0.2136	0.3158
γ (″)	0.0036	0.295	0.1852	0.3563	0.0025	0.2461	0.1637	0.3353

**Table 4:** Simulation results of different heading angles.

Parameter	0°	30°	60°	90°	120°	150°	180°
$\varepsilon_{x}$ (°/h)	0.0001	0.0002	0.0003	0.0004	0.0002	0.0003	0.0004
$\varepsilon_{y}$ (°/h)	0.0002	0.0002	0.0003	0.0004	0.00005	0.0004	0.0005
$\varepsilon_{z}$ (°/h)	−0.0002	0.0003	−0.0003	0.0003	−0.0003	0.0002	−0.0003
$\nabla_{x}$ (μg)	−0.8282	0.8047	−0.7621	0.678	−0.6723	0.9058	−1.1413
$\nabla_{y}$ (μg)	−0.4469	0.4493	−0.4953	0.4189	−0.4932	0.5359	−0.7016
$\nabla_{z}$ (μg)	0.2836	0.003	0.4408	0.5425	0.0634	0.3509	0.8046
$k_{ax}$ (ppm)	0.2310	0.2100	0.1528	0.3561	0.1059	0.4795	0.2095
$k_{ay}$ (ppm)	−0.1640	−0.1014	−0.1108	−0.1751	−0.2135	0.1915	−0.1813
$k_{az}$ (ppm)	−0.1199	−0.1785	−0.1568	−0.1210	−0.1812	−0.1964	−0.2170
$k_{gx}$ (ppm)	0.0834	0.5543	−0.7975	1.1018	0.9184	−0.1006	−0.9222
$k_{gy}$ (ppm)	0.5652	0.4680	0.2568	0.4750	0.5810	−0.7252	1.1168
$k_{gz}$ (ppm)	0.0618	0.3218	0.0911	0.6660	0.2012	0.3106	−0.6793
$S_{gxy}$ (″)	−0.1669	0.1989	−0.1252	0.2270	−0.1389	0.1585	0.5027
$S_{gxz}$ (″)	0.2890	0.3614	0.1423	0.5180	0.0331	0.3029	0.5712
$S_{gyx}$ (″)	−0.3021	−0.3992	−0.2673	−0.5448	−0.3001	−0.5239	−0.7781
$S_{gyz}$ (″)	0.1665	0.1887	0.3553	0.7222	0.3628	0.6499	0.9026
$S_{gzx}$ (″)	0.1506	0.2519	0.2645	0.4042	0.1984	0.2353	0.5648
$S_{gzy}$ (″)	−0.2413	−0.1235	−0.3786	−0.4283	−0.4807	−0.4395	−0.5220
$S_{ayx}$ (″)	0.3448	0.8615	0.4011	0.7038	0.3126	0.5732	0.9560
$S_{azx}$ (″)	0.1160	0.3993	0.3379	0.3820	0.5392	0.3255	0.9529
$S_{azy}$ (″)	0.4830	0.3893	0.4517	0.4436	0.3257	0.3720	0.7392
α (″)	0.0563	0.0611	0.2052	0.2174	0.2938	0.2404	0.3120
β (″)	−0.1074	0.5557	−0.1808	0.3342	−0.3589	0.5153	−0.7725
γ (″)	0.0307	0.0573	0.0952	0.0495	0.3831	0.6351	0.6807

**Table 5:** Specifications of the dual-axis rotational inertial navigation system.

Characteristics	Description
Sampling frequency	200 Hz
Gyro-constant drift	≤ 0.005 deg/h
Gyro-stochastic drift	≤ 0.001 deg/sqrt(h)
Gyro-scale factor repeatability	≤ 50 ppm (1σ)
Gyro-constant drift repeatability	≤ 0.001 deg/h (1σ)
Gyro installation error	≤ 0.001 rad
Accelerometer constant bias	≤ 10 μg
Accelerometer-scale factor repeatability	≤ 50 ppm (1σ)
Accelerometer constant bias repeatability	≤ 10 μg (1σ)
Accelerometer installation error	≤ 0.001 rad
Rotation angle accuracy	0.5″
Rotation velocity accuracy	0.7″/s

**Table 6:** Comparison of the velocity and position errors.

Parameter		LSEAVC-KF	LSOAVC-KF
Ve error (m/s)	RMSE Maximum	0.0595 0.0870	0.1002 0.1515
Vn error (m/s)	RMSE Maximum	0.0403 0.0661	0.0706 0.1092
Pe error (m)	RMSE Maximum	110.0472 175.9277	130.3365 216.0348
Pn error (m)	RMSE Maximum	40.4820 100.6326	82.8674 185.4080

## Data Availability

The data that support the findings of this study are available from the corresponding author upon reasonable request.
